# Synthesis and Characterization of Fluorite-Type La_2_Ce_2_O_7_ Plasma Sprayable Powder for TBCs Application

**DOI:** 10.3390/ma15114007

**Published:** 2022-06-05

**Authors:** Ivana Parchovianská, Milan Parchovianský, Beáta Pecušová, Ondrej Hanzel, Amirhossein Pakseresht

**Affiliations:** 1 Centre for Functional and Surface Functionalised Glass, Alexander Dubček University of Trenčín, Študentská 2, 911 50 Trenčín, Slovakia; milan.parchoviansky@tnuni.sk (M.P.); beata.pecusova@tnuni.sk (B.P.); amir.pakseresht@tnuni.sk (A.P.); 2 Institute of Inorganic Chemistry, Slovak Academy of Sciences, Dúbravská Cesta 9, 845 36 Bratislava, Slovakia; ondrej.hanzel@savba.sk

**Keywords:** La_2_Ce_2_O_7_, powder, Pechini sol-gel, solid-state synthesis, flame synthesis

## Abstract

This work focuses on the fabrication of lanthanum cerate (La_2_Ce_2_O_7_, LC) powders via two chemical routes: modified Pechini sol-gel method and solid-state synthesis. The synthesized LC powders were heat treated in the temperature range of 1000–1400 °C for 6 h and investigated as a material for thermal barrier coating (TBC) applications. For this purpose, the powder morphology, chemical composition, crystal structure and thermal stability were studied. Scanning electron microscopy (SEM) of the synthesized powders revealed an agglomerated structure consisting of fine and uniformly distributed grains. Energy-dispersive X-ray spectroscopy (EDXS) indicated that the chemical compositions of the LC powders were similar to the stoichiometric ratio of La_2_Ce_2_O_7_. A cubic fluorite structure was observed by X-ray diffraction analysis (XRD) after calcining the LC powder prepared by solid-state synthesis at 1300 °C. In contrast, there was always a fluorite structure in the LC powder synthesized by the Pechini sol-gel method after heat treatment over the entire temperature range. The thermal behavior of the LC powders was analyzed by differential scanning calorimetry (DSC) and thermogravimetric analysis (TG) in the temperature range of 25–1300 °C. Neither an obvious mass change nor a visible energy change was observed within the tested temperature range, indicating high phase stability of the LC powder and its suitability for TBC applications. Spheroidization on the prepared LC powders was also investigated, revealing that powder size and morphology had a significant impact on the spheroidization efficiency.

## 1. Introduction

Recently, there has been a considerable interest in the synthesis and structural characterization of rare earth metal (RE)-doped cerates due to their outstanding catalytic, electrical and mechanical properties, as well as their improved chemical stability and ionic conductivity [1,2]. Such properties make these materials promising candidates for many applications, such as ionic conductors in solid oxide fuel cells, oxygen sensors, catalyst carriers, hydrogen separation membranes or thermal barrier coatings (TBCs) [3,4,5,6,7]. TBCs have been applied in gas turbines for decades to decrease the surface temperature of hot sections of the metallic components and to protect them from combustion environments. The choice of TBC materials is limited by some basic requirements, such as a high melting point, low thermal conductivity, thermal expansion match with the metallic substrate, no phase transformation during service, chemical stability and good adhesion to the underlying metallic substrate [2,6].

Among various RE-doped cerates, La_2_Ce_2_O_7_ (LC) is one of the most promising TBC materials for high-temperature applications due to its high phase stability and ability to withstand high temperatures (>1250 °C) [6,8]. Moreover, LC exhibits a higher thermal expansion coefficient (CTE), lower thermal conductivity and better calcium–magnesium–aluminum–silicate (CMAS) corrosion resistance than the conventional TBC with an yttria-stabilized zirconia (YSZ) top layer [2,7,8,9,10]. La_2_Ce_2_O_7_ is a solid solution of La_2_O_3_ in CeO_2_ with a defect fluorite structure [6]. Oxides with a general formula of A_2_B_2_O_7_ crystallize to ordered pyrochlore and disordered fluorite-type structures. The disordered La_2_Ce_2_O_7_ structure is acquired by replacing half of the Ce^4+^ cations with La^3+^, while one-eighth of the O anions are removed through random selection due to charge compensation. Cubic fluorite structures like this have a desired stoichiometry, with each cation site being occupied by 0.5 Ce and 0.5 La atoms on average, and each anion site is on average occupied by 0.875 O atoms [11]. Bae et al. [3] reported that the CeO_2_ fluorite structure is preserved for La concentrations up to x = 0.40 (in La_2_Ce_2_O_7_, x = 0.50), and only for higher La concentrations will the pyrochlore arrangement of the cations occur. It has been also observed that the disordered fluorite structure is favorable when the cation radii ratio *r_A_*/*r_B_* is lower than 1.46 [12].

In the past few years, several methods have been employed for the synthesis of RE-doped cerates, such as conventional solid-state synthesis [12,13], co-precipitation route [1,14], the hydrothermal method [15,16], sol-gel [17,18], the molten salt method [19] and the citrate–nitrate combustion method [20]. Some of these methods are expensive and require multiple steps. Furthermore, in the case of La_2_Ce_2_O_7_ synthesis by the conventional co-precipitation and hydrothermal methods, water is used as a solvent, which could result in a high possibility of forming hard agglomerates [21]. The compounds prepared by the traditional solid-state route require long, high-temperature treatments, and micron-sized particles are formed. Nano-sized lanthanum cerate with good powder homogeneity and a low crystallization temperature can be prepared by alkoxide-based sol-gel and citrate methods. Many research articles have been also focused on investigating the structure and properties of LC. For example, Hongsong et al. [17] studied the photo-catalytic properties of La_2_Ce_2_O_7_ powder synthesized by a sol-gel method. The optical properties of La_2_Ce_2_O_7_ nano-powders were investigated in the work of Khademinia and Behzad [16]. Wang et al. [22] analyzed the crystal growth and sintering behavior of La_2_Ce_2_O_7_ nanocrystals. Ma et al. [9] investigated the phase stability and thermal expansion coefficients of La_2_Ce_2_O_7_ solid solutions. In recent decades, considerable efforts have been dedicated to improving the performance of LC thermal barrier coatings. For instance, Dehkharghani et al. [23] devoted their work to improving the thermal shock resistance and fracture toughness of La_2_Ce_2_O_7_ thermal barrier coatings. Zhang et al. [24] reported the mechanical and thermal cycling performance of YSZ-toughened La_2_Ce_2_O_7_ composite thermal barrier coatings. 

In the present study, pure La_2_Ce_2_O_7_ powders with a fluorite structure were fabricated by both solid-state synthesis and a modified Pechini sol-gel method [25]. This method has been used as an alternative to the conventional sol-gel method. The main advantage of the Pechini sol-gel method is its simplicity and low cost. Furthermore, the metallic ions are immobilized in a rigid polymer network, which ensures their homogeneous dispersion in the polymer network without precipitation or phase segregation. This process provides thorough control over the product stoichiometry, even for more complex oxide powders. In addition, the Pechini sol-gel route allows the reactant cations to mix at the atomic scale, leading to an increase in reaction rate and a lowered synthesis temperature [26,27].

Usually, LC powder with a desired structure is difficult to obtain commercially; consequently, it must be synthesized in the laboratory and then transformed to a plasma sprayable powder for producing TBCs. To ensure good deposition efficiency and improve the coating properties, the density, shape, size and flow characteristics of such particles should be controlled. Moreover, particles with spherical morphology are preferred over particles with an irregular shape. Spray drying, plasma processing and gas atomization procedures are commonly used for fabricating spherical particles [2,28,29,30]. In this study, we attempted made to produce nonporous La_2_Ce_2_O_7_ spherical particles with flame synthesis as a new method.

In this work, La_2_Ce_2_O_7_ powders with a fluorite structure were synthesized via two chemical processes (solid-state synthesis and modified Pechini sol-gel method) to compare the morphologies and study their crystal structure and thermal stability. The purpose of this work is to explore more favorable method for preparing LC powders and to investigate for the first time the spheroidization process of LC particles by flame synthesis.

## 2. Materials and Methods

### 2.1. Powder Synthesis

**Figure 1:**
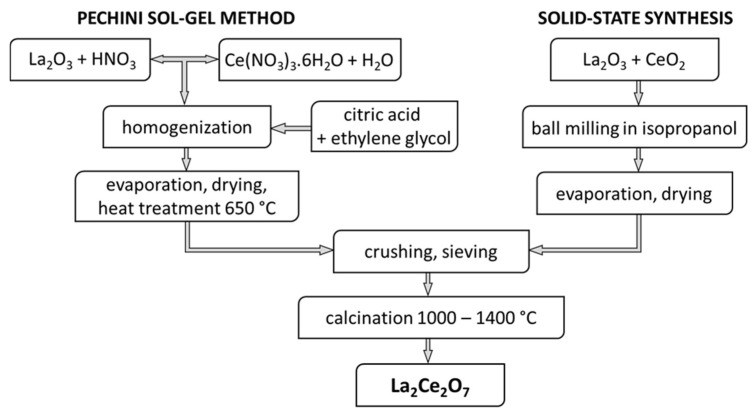
Scheme of obtaining LC powders by Pechini sol-gel method and solid-state synthesis.

In this work, LC powder was synthesized by two chemical processes: modified Pechini sol-gel method and solid-state reaction, as illustrated in Figure 1. For the Pechini sol-gel route, La_2_O_3_ (99.95%, Alchimica, Praha, Czech Republic) was first dissolved in concentrated nitric acid to form its respective nitrate solution. Next, the calculated amount of Ce(NO_3_)_3_.6H_2_O (99.95%, Treibacher Industrie AG, Althofen, Austria) was dissolved in deionized water and added to the nitrate solution. Subsequently, an aqueous solution of citric acid and ethylene glycol with a 1:1 molar ratio was added to the resultant mixture dropwise and heated in an oil bath under continuous stirring at ~85 °C for 2 h. The molar ratio of citric acid/cerium was 2:1. The solution was then slowly evaporated until a solid porous mass was obtained. Finally, the product was dried in an oven at 120 °C for 12 h, crushed and then heat treated at 650 °C in a muffle furnace for 12 h to burn out the organic compounds.

For the solid-state reaction procedure, stoichiometric amounts of commercial binary oxides La_2_O_3_ and CeO_2_ (both 99.95% purity, Alchimica, Praha, Czech Republic) were mixed and ball-milled with zirconia balls in isopropanol for 24 h. The suspension was then dried by continuous stirring to remove the excess alcohol. For both processes, i.e., modified Pechini sol-gel method and solid-state reaction, the obtained LC powders were sieved through a 40 μm analytical sieve and calcined in an electric furnace at temperatures of 1000 °C, 1100 °C, 1200 °C, 1300 °C and 1400 °C for 6 h. The powders are labeled according to the applied synthesis route, i.e., LC-SG and LC-SS represent the Pechini sol-gel and solid-state reaction route, respectively.

### 2.2. Characterization Methods

The crystalline phases of the prepared LC powders calcined at different temperatures were identified by X-ray powder diffraction (XRD, PANalytical Empyrean DY1098 (Panalytical, BV, Almelo, The Netherlands)) using a Cu anode and an X-ray wavelength of λ = 1.5405 Å over 2θ angles of 10–80°. Diffraction records were evaluated using HighScore Plus (v. 3.0.4, Panalytical, Almelo, The Netherlands) with the use of the PDF4 database. The mean crystallite size of the prepared powders was calculated by Scherrer’s Equation (1):

(1) $$D=\frac{0.89\lambda }{\beta cos\theta },$$

where *D* is the crystallite size, *λ* is the X-ray wavelength, *β* is the peak width at half of the maximum intensity and *θ* is the diffraction angle. Raman spectra of the LC powders heat treated at 1400 °C for 6 h were recorded in the range of the Raman shift (100–800) cm^−1^ by a RENISHAW inVia Reflex Raman spectrometer (RENISHAW, Wotton-under-Edge, England, UK). The morphology of the produced powders was examined in detail by scanning electron microscopy (SEM, JEOL JSM 7600 F, JEOL, Tokyo, Japan). The chemical composition of the powders was determined by energy-dispersive X-ray spectroscopy (EDXS, Oxford Instruments, Abingdon, UK). The thermal stability studies of the powders were conducted using thermogravimetric analysis and differential scanning calorimetry (TG/DSC, Netzsch STA 449 F1 Jupiter, NETZSCH-Gerätebau GmbH, Selb, Germany) in the temperature range of 25–1300 °C with a heating rate of 10 °C/min. A sample weight of ≈13 mg was used for the TG/DSC experiments.

### 2.3. Spheroidization of LC Powder

**Figure 2:**
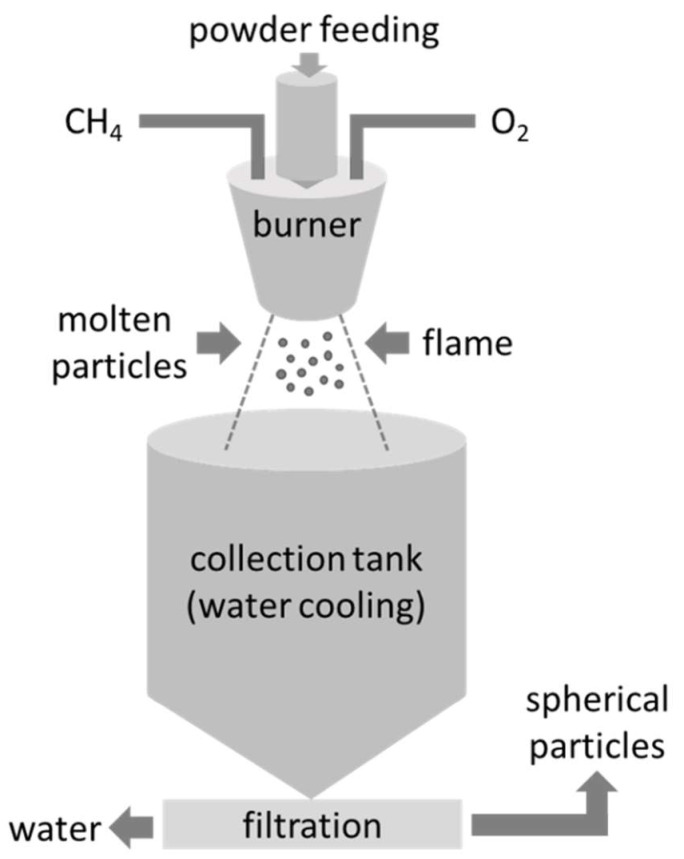
The schematic of preparing spherical LC particles by flame synthesis.

To increase the powder density and decrease the porosity, the LC particles were spheroidized by flame synthesis. The laboratory equipment for the flame synthesis is located at the FunGlass Centre (Trenčín, Slovakia) and was approved as a utility model [31]. A schematic drawing with descriptions of the individual parts of the used device is shown in Figure 2. The prepared LC powders were fed into a high-temperature flame (CH_4_/O_2_, T ~2200 °C) using a vacuum powder feeder. The molten particles were quenched in deionized water and collected in a container. Then, the spheroidized particles were micro-filtered through a ceramic filter (porosity < 0.3 µm) located below the collection tank and the products were dried overnight at ~120 °C and calcined at 650 °C for 4 h to remove any organic residue. The overall efficiency of the laboratory device for flame synthesis is in the range of 75–85%. The investigation of the spherical particles was conducted with the use of SEM/EDXS. The SEM images were also used to evaluate the size and size distribution of the spherical particles using the Lince (TU Darmstadt, Darmstadt, Germany) software for image analysis. The particle size distribution was obtained by the analysis of ten different SEM images recorded at a 500× magnification, measuring the diameters of at least 200 spherical particles. For a more detailed examination of the microstructure, the samples were cold mounted in polymeric resin and carefully polished (EcoMet 300, Buehler, Leinfelden-Echterdingen, Germany) to prepare cross-sections. XRD was used to investigate the phase composition of the spheroidized particles.

## 3. Results and Discussion

### 3.1. Characterization of Prepared Powders

**Figure 3:**
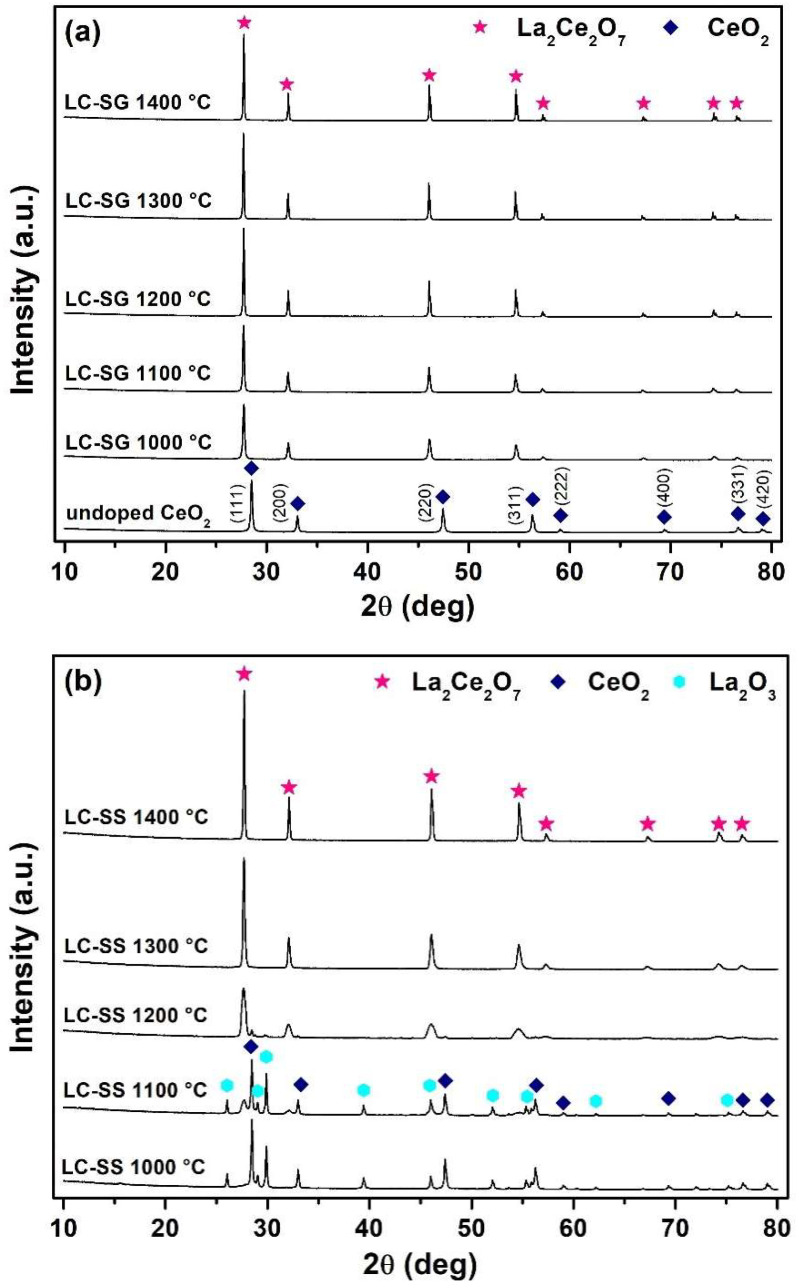
XRD patterns of the LC powders calcined at different temperatures: (**a**) LC-SG and (**b**) LC-SS.

The XRD is an important analytical technique to investigate the structure of La_2_Ce_2_O_7_. The differences between fluorite and a typical pyrochlore pattern are small and are mainly differentiated by the presence of characteristic low-intensity pyrochlore peaks located at 36.9° and 44.5° 2θ [15]. XRD patterns of LC-SG and LC-SS powders after heat treatment at various temperatures are shown in Figure 3. For comparison, the XRD pattern of a pure CeO_2_ powder is also included in Figure 3a. Evidently, XRD patterns of all LC-SG samples (Figure 3a) match well with the cubic fluorite phase of CeO_2_, as the XRD records show eight clear peaks that can be well designated to the (111), (200), (220), (311), (222), (400), (331) and (420) lattice planes of the fluorite structure [13]. Diffraction peaks corresponding to unreacted La_2_O_3_ or CeO_2_ were not detected in the XRD patterns of the LC-SG powder, confirming the formation of La_2_Ce_2_O_7_ solid solution with high phase purity. Moreover, the intensities of the XRD peaks corresponding to the La_2_Ce_2_O_7_ phase increased with increasing temperature, indicating the high crystallinity of the powder.

For the LC-SS powders, additional peaks corresponding to La_2_O_3_ were observed in the XRD patterns of the powder heat treated up to 1200 °C (Figure 3b). It can be seen that the La_2_Ce_2_O_7_ phase starts to form from 1100 °C. In contrast, the intensities of the diffraction peaks corresponding to La_2_O_3_ and CeO_2_ gradually decreased with increasing temperature. When the temperature reached 1300 °C, a single La_2_Ce_2_O_7_ phase was formed and the peaks belonging to La_2_O_3_ and CeO_2_ totally disappeared. Moreover, the diffraction peaks of the La_2_Ce_2_O_7_ phase became sharper with increasing temperature, indicating a crystallite size increase. The diffraction peaks of the La_2_Ce_2_O_7_ phase in both powders are shifted to lower angles compared to undoped CeO_2_ powder because of the larger radius of La^3+^ (0.103 Å) than Ce^4+^ (0.087 Å) [32]. In both cases (i.e., LC-SG and LC-SS), no secondary pyrochlore peaks are visible in the XRD spectra. This is consistent with the XRD spectra of La_2_Ce_2_O_7_ generally presented in literature [9,14,33,34]. It was also reported that the LC fluorite structure remains stable, even at very high temperatures [6,9]. The results presented here also indicate that the Pechini sol-gel method synthesis temperature for La_2_Ce_2_O_7_ was lowered from the 1300 °C of the solid-state reaction to 1000 °C. The fluorite structure of LC powder prepared by solid-state synthesis was also identified at 1300 °C in the work of Dehkharghani et al. [13,23].

**Figure 4:**
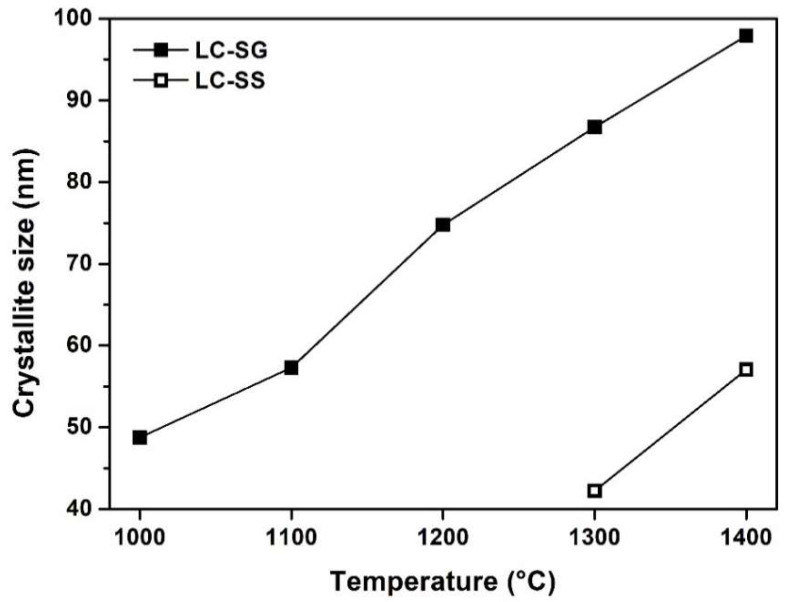
The average crystallite size of LC powders as a function of calcination temperature.

The crystallite size of the prepared LC powders was calculated using Scherrer’s Equation (1). Figure 4 shows the relationship between the calcination temperature and the average crystallite size of LC powders prepared by different methods. It is evident that as the calcination temperature increased, the crystallite size increased due to the sintering effect. This was also shown in the intensities of the diffraction peaks. As shown in Figure 4, the average crystallite size of LC-SG powder calcined at different temperatures from 1000 °C to 1400 °C varied from 48 nm to 98 nm. The calculated crystallite size for LC-SS is 42 nm for the sample calcined at 1300 °C and 57 nm for the sample calcined at 1400 °C. As can be noted from Figure 4, the LC-SS powder exhibited a smaller crystallite size compared with that of the LC-SG powder calcined at the same temperature. The results indicate that the synthesis route affects the crystallite size of the fluorite-type La_2_Ce_2_O_7_. Wang et al. [22] synthesized LC nanoparticles at 1100 °C via the hydrothermal method using polyethyleneglycol as a surfactant. It was also shown that when increasing the calcination temperature from 700 °C to 1300 °C, the average crystallite size of La_2_Ce_2_O_7_ varied from approximately 11 nm to 60 nm. The fluorite structure of LC was also successfully synthesized at 800 °C by a sol-gel method, with the average crystallite size of LC powders ranging from 10 nm to 30 nm [17]. A smaller crystallite size was obtained by the co-precipitation route using triethylamine [1]. The cubic fluorite structure of LC was observed after heating the sample at 600 °C and 900 °C for 3 h, while the calculated crystallite sizes were 5 nm and 28 nm, respectively.

The lattice parameters of the produced LC samples were determined by XRD using the most intense (111) line, and the obtained results are presented in Table 1. Compared to the lattice parameter for undoped CeO_2_ (*a* = 0.54187 nm), there is an increase in the cell parameter in both LC samples (see Table 1). This can be attributed to lattice expansion during partial substitution of Ce^4+^ ions with larger La^3+^ ions [35]. No influence of the calcination temperature on the lattice parameters of the LC-SG and LC-SS samples was observed in the present study. In general, the small crystallite size and stable cell parameter during the high temperature treatment implies that the investigated material has suitable thermal stability [35]. 

**Figure 5:**
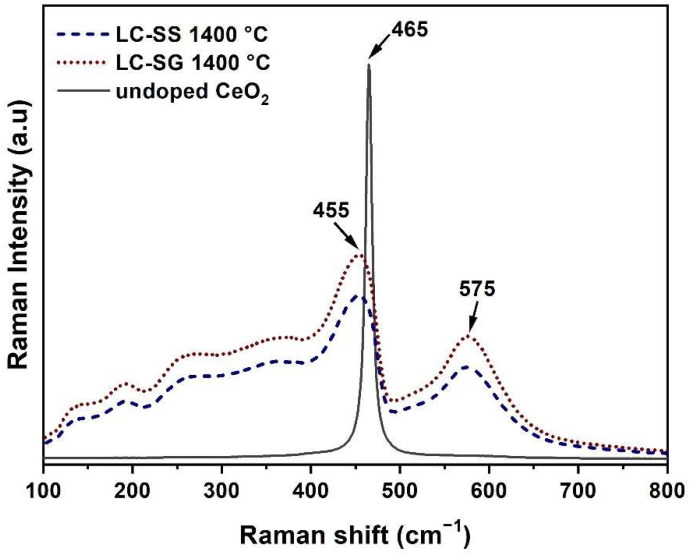
Raman spectra of the LC powders calcined at 1400 °C.

Raman spectroscopy has proven to be a useful analytical technique, providing information about the crystalline structure of materials [36,37,38]. To confirm the solid solution phase, the LC-SG and LC-SS powders heat treated at 1400 °C were investigated by Raman spectroscopy, as shown in Figure 5. For comparison, the Raman spectrum of pure CeO_2_ is also included in Figure 5. According to the literature [39], the ideal fluorite structure has only one allowed Raman active mode (F_2g_). This mode correlates with the symmetric vibration of oxygen atoms around each cation. In the case of undoped CeO_2_, there is only a single peak centered at ~465 cm^−1^ and it corresponds to the F_2g_ Raman band from the space group Fm3m of a cubic fluorite structure [17]. As illustrated in Figure 5, the synthesized LC-SG and LC-SS powders have similar patterns: Raman spectra show one dominant band at ~455 cm^−1^, one broader band at ~575 cm^−1^ and four weak bands at lower frequencies. Remarkably, the F_2g_ modes at ~455 cm^−1^ are shifted to a lower frequency and become broader and more asymmetric. This is a common effect of rare earth doping on the F_2g_ mode [35,39]. Moreover, the intensity of the peak at ~455 cm^−1^ is higher for LC-SG compared to that of the LC-SS. The broad bands at higher frequencies, i.e., at ~575 cm^−1^, were assigned to oxygen vacancies as a result of La^+3^ incorporation into a fluorite type CeO_2_ matrix [15]. The weak bands at low frequencies in the Raman patterns of LC powders can be attributed to second-order scattering and forbidden acoustic modes caused by defects in the structure [12,36]. The results of Raman spectroscopy are consistent with the XRD results, as they prove that both LC powders still maintain the fluorite structure up to 1400 °C.

**Figure 6:**
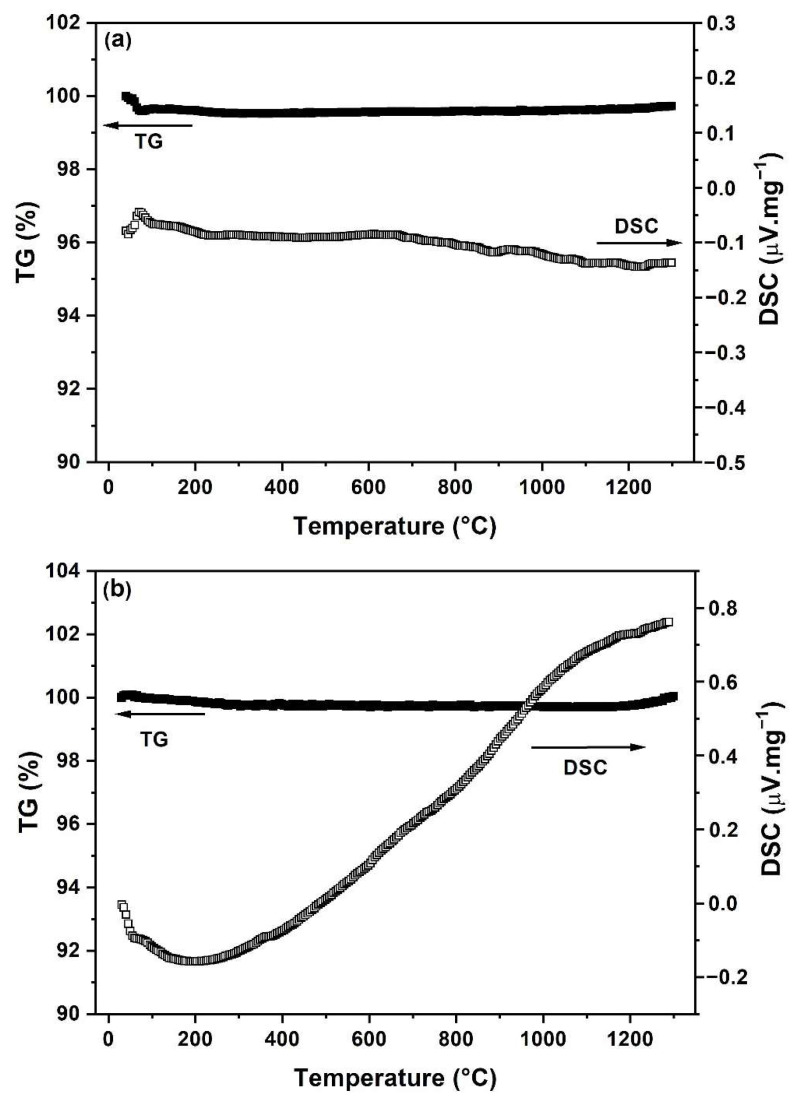
TG and DSC curves of the prepared powders: (**a**) LC-SG, (**b**) LC-SS.

To evaluate the thermal behavior and phase stability of the prepared powders, TG/DSC records were measured from room temperature to 1300 °C, as shown in Figure 6. To investigate the possible phase transformations, the LC-SG and LC-SS powders calcined at 1000 °C and 1300 °C, respectively, were used for thermal analysis. As can be seen in Figure 6, both LC-SS and LC-SG powders exhibit high phase stability since neither an endothermic peak nor an exothermic peak were observed in the temperature range of 100–1300 °C. In the DSC curve of LC-SG powder (Figure 6a), only a small peak occurring at ~75 °C is observed and corresponds to a release of absorbed moisture. These results also prove that both prepared powders still preserve the fluorite structure within the experimental temperatures. In the case of the TG curve for LC-SS (Figure 6b), there is no obvious mass loss in the tested temperature range. For the TG curve of LC-SG, the negligible mass loss of 0.26% was observed because of the evaporation of physically adsorbed water and decomposition of residual nitrates and organic compounds.

**Figure 7:**
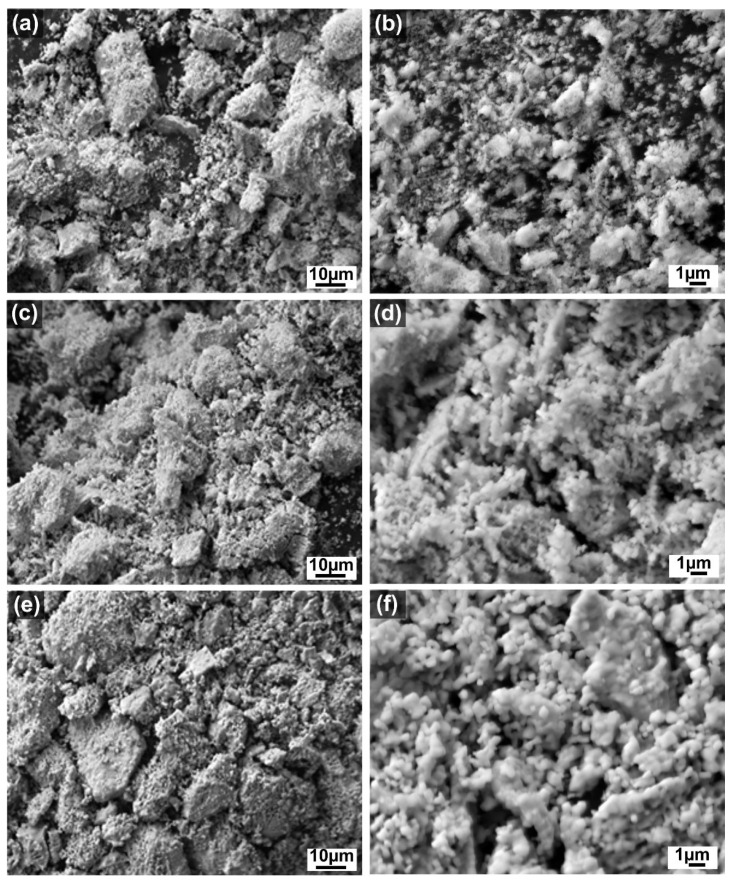
SEM images of the LC-SG powders calcined at different temperatures: (**a**) 1000 °C; (**b**) 1000 °C—higher magnification; (**c**) 1200 °C; (**d**) 1200 °C—higher magnification; (**e**) 1400 °C; (**f**) 1400 °C—higher magnification.

**Figure 8:**
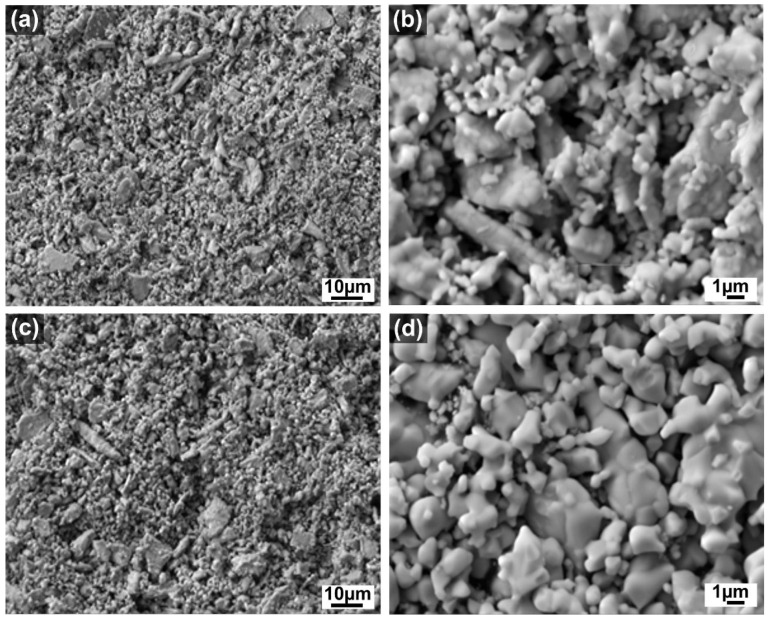
SEM images of the LC-SS powders calcined at different temperatures: (**a**) 1300 °C; (**b**) 1300 °C—higher magnification; (**c**) 1400 °C; (**d**) 1400 °C—higher magnification [41].

SEM analysis was employed to obtain important information about the morphology and size of the produced LC powders. Figure 7 and Figure 8 show the morphology of the LC powders calcined up to 1400 °C under two different magnifications. As shown in the microstructural images (Figure 7a–f), the morphology of the sieved LC-SG powder is block-shaped and the particles are made of agglomerated, small primary particles. The irregular and angular shape of the grains are a result of the crushing process. According to the magnified microstructural images (Figure 7b,d,f), an approximate estimation of the size of the primary nanoparticles in LC-SG powder is less than 200 nm. The size of the nanocrystals became larger as a function of calcination temperature. This agrees with the crystallite size estimated from Scherrer’s equation. When the calcination temperature reached 1400 °C, a kind of sintering process took place, and the nanocrystals formed more aggregates. 

Figure 8a–d illustrates the SEM images of the as-prepared LC-SS powder calcined at 1300 °C and 1400 °C. SEM examination of the LC-SS powder also revealed an agglomerated structure consisting of finely and uniformly distributed irregular particles that agglomerated to form larger particles, similar to LC-SG powders. As can be seen in the magnified microstructural image (Figure 8d), heat treatment up to 1400 °C resulted in powder sintering and grain growth. Therefore, determining the size of the primary particles was difficult. It is evident that different synthesis route influenced the particle size and morphology of the prepared material. The obtained SEM images indicate that the homogeneity and size distribution of the nanoparticles are better for LC-SG powder. At a calcination temperature of 1400 °C, the obtained LC-SS nanocrystals showed more irregularly sized blocks made of aggregated and sintered particles. This is due to the smaller size of the nanocrystals compared to LS-SG powder. 

Wang et al. [19] used SEM analysis to obtain direct information about the size and structure of the LC nano-powders produced by the molten salt method. The as-obtained LC powders were found to be agglomerated and composed of small particles with average size ranging from 50 nm to 80 nm. Joulia et al. [40] reported that the LC powder prepared by citrate route was composed of fine and uniform 50–100 nm particles, but the sintering effect led to a strong grain growth at 1400 °C. Liu et al. [20] found that both the agglomerate and dispersive nanoparticles exist in the LC powder synthesized by citrate-nitrate combustion method, and the average grain size was less than 100 nm. Hongsong et al. [17] studied the micro-morphology of the LC powders prepared by the sol-gel method. They found that the synthesized powders have a relatively uniform size and exhibit a certain degree of agglomeration caused by large surface energy. Generally, high-temperature treatment causes gradual sintering and crystallite growth, leading to a loss of surface area. It is well known that smaller particles have higher surface energy that in turn provides a greater driving force for sintering [19]. However, the resistance of the rare earth-doped cerates to thermal sintering is significant compared to that of pure ceria [35].

EDXS analysis was performed in order to verify the elemental composition of the prepared LC powders. The EDXS results of the LC powders calcined at 1400 °C for 6 h are presented in Table 1. Both LC powders exhibit only a small deviation from the theoretical composition of La_2_Ce_2_O_7_. The deviation was the highest for oxygen, which measured lower than the theoretical value. The stoichiometric atomic ratios of La:Ce:O are 1:1.01:3.34 for LC-SS and 1:1.02:3.41 for LC-SG, compared to the original ratio 1:1:3.5. 

### 3.2. Spheroidization of LC Powder

**Figure 9:**
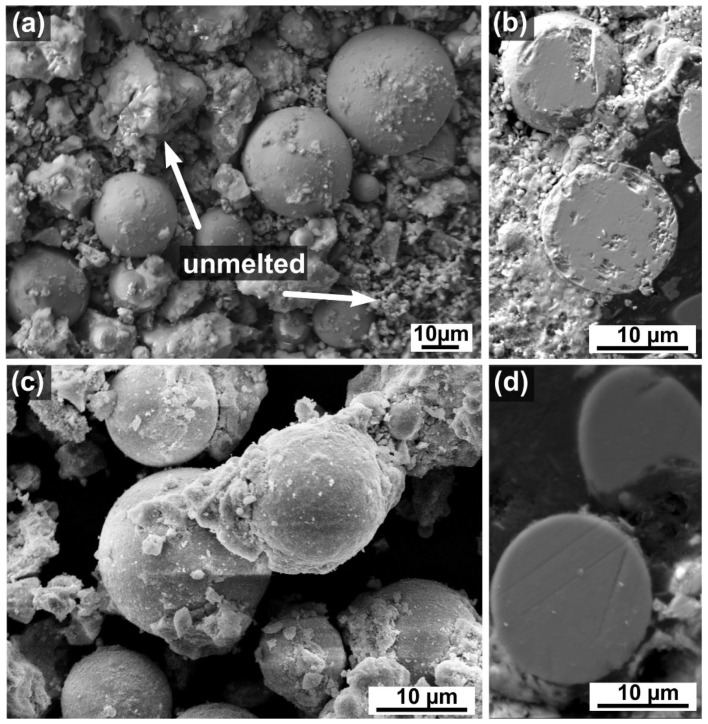
SEM images of spheroidized LC particles: (**a**) LC-SS surface view, (**b**) LC-SS cross-section, (**c**) LC-SG surface view, (**d**) LC-SG cross-section.

Figure 9 presents the microstructural images of LC-SG and LC-SS powders processed by flame synthesis. As is obvious from Figure 9a, a significant fraction of un-melted powder with an irregular structure is observed after flame synthesis of the LC-SS powder. Only a small amount of spherical particles was detected. Moreover, some defects and small pores are visible in the cross-section of the spherical particles (Figure 9b). Such low spheroidization could be attributed to the fact that the agglomerated LC-SS particles stuck to one other as well as to the walls of the powder feeder, making it difficult for them to enter the burner and reach the high-temperature flame. As shown in Figure 9c, the content of un-melted particles decreased when using LC-SG powder, and the particle morphologies changed from irregular to almost fully spheroidized. Such spherical morphology could increase the deposition efficiency in TBCs. Moreover, the amount of spherical particles is larger than in the case of LC-SS powder spheroidization. The presence of un-melted and semi-melted particles in both powders might also be due to an insufficient flame temperature for complete re-melting of the powder or a short retention time in the flame. As can be seen from a cross-sectional image of the spheroidized LC-SG powder (Figure 9d), the spherical particles do not exhibit the presence of defects or intraparticle pores, confirming a density increase and the melting of the powder. However, the parameters of flame synthesis need to be further optimized to increase the spheroidization efficiency and to achieve a fully re-melted LC powder.

**Figure 10:**
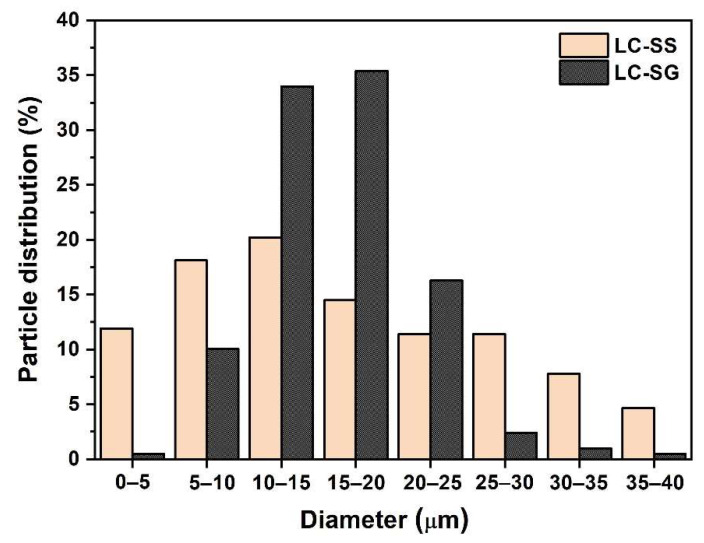
Particle size distribution of the LC spherical particles.

Granulometry of the LC spherical particles (Figure 10) was determined via computer image analysis of the SEM micrographs. The LC-SG spherical particles yielded two main fractions with diameters in the intervals of 10–15 μm (~34%) and 15–20 μm (~35%). Only a small fraction of spherical particles showed larger and smaller diameters. As for LC-SS, the particle sizes are evenly distributed across the entire size range, with the main fraction being between 5 and 15 μm (~38% total).

The EDXS results showed that the spheroidization process of LC powder by flame synthesis led to a considerable deviation from stoichiometry due to loss of lanthanum. The EDXS analysis of the LC-SG spherical particles showed the chemical composition of the material is 60.3 at % O, 24.3 at % Ce and 15.4 at % La. Similar results have been reported by Praveen et al. in the case of plasma-transferred arc synthesis of LC powder [29]. Loss of lanthanum has also been observed by Wen Ma et al. while investigating the thermal cycling behavior of LC coatings obtained by EB-PVD [8]. The loss of La_2_O_3_ could be effectively reduced by increasing the size of the powder particles for flame synthesis or by adding an excess of La_2_O_3_ into the starting powder to prepare nearly stoichiometric La_2_Ce_2_O_7_ spherical particles. However, several authors [2,42] reported that partial decomposition of La_2_Ce_2_O_7_ and a loss of CeO_2_ occurred during the preparation of TBCs by plasma spraying or electron beam physical vapor deposition due to the different vapor pressures of La_2_O_3_ and CeO_2_. This led to a compositional deviation of the sprayed LC coatings from the original LC powder. Because of this, in further work, it will be necessary to optimize and carefully design the chemical composition of the original powders in order to obtain LC coatings with a near stoichiometric composition. 

**Figure 11:**
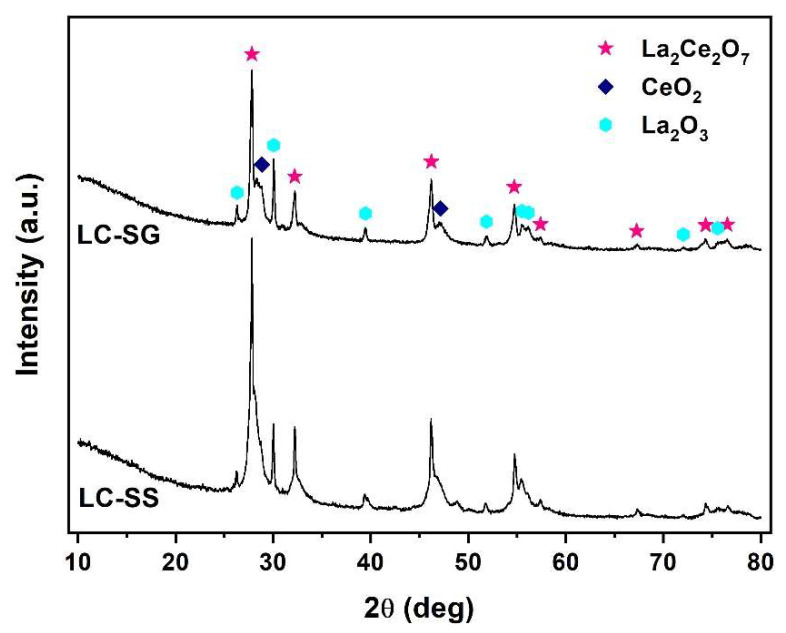
XRD patterns of LC particles processed by flame synthesis.

The XRD profiles of the LC-SG and LC-SS samples processed by flame synthesis are presented in Figure 11. XRD analysis of both spheroidized LC samples revealed that the fluorite structure is still present; however, a few additional weak peaks corresponding to La_2_O_3_ and CeO_2_ are also observed. The presence of these peaks can be attributed to partial decomposition of the powder material and phase separation of La_2_O_3_ from the La_2_Ce_2_O_7_ solid solution during flame synthesis. This is also supported by the presence of CeO_2_ in the diffraction patterns due to the deviation in the La/Ce ratio of the spheroidized LC samples with respect to stoichiometric LC. These results indicate that both LC powders did not preserve their fluorite phase structure during flame synthesis. Deeper research on the spheroidization process of LC particles and the effect of their morphology on the final microstructure and properties of plasma-sprayed TBCs is in progress and will be published separately. Future work will also focus on the preparation and characterization of LC-YSZ composites as potential materials for TBCs. The chemical reactivity of the LC and YSZ, sintering behavior and the mechanical and thermal properties of the LC-YSZ bulk samples will be investigated at high temperatures and pressures via hot-press experiments.

## 4. Conclusions

The results presented in this paper provide a basic understanding of the structure of La_2_Ce_2_O_7_ prepared by two different methods, namely, the modified Pechini sol-gel method and solid-state synthesis. The cubic fluorite structure of the LC-SS powder was observed by XRD after heat treatment at 1300 °C. In contrast, there was always a single fluorite structure in the LC-SG powder after heat treatment over the entire temperature range of 1000–1400 °C. Therefore, it can be concluded that the synthesis temperature was lowered from 1300 °C to 1000 °C by the modified Pechini sol-gel method. SEM examination showed that the as-obtained La_2_Ce_2_O_7_ powders were composed of agglomerates formed of nano-grains with crystallite sizes ranging from 42 to 98 nm, depending on the synthesis method and calcination temperature. However, more homogeneity in the size and shape of the grains was observed for the LC-SG sample. As shown by EDXS, both powders have a chemical composition close to the stoichiometric composition of La_2_Ce_2_O_7_. A detailed review of the Raman spectra confirmed that both LC-SS and LC-SG have a fluorite structure that remains stable after calcination at 1400 °C. The investigations in this work indicate that the prepared LC powders are promising candidates for TBC applications and that the Pechini sol-gel process is a simple way to prepare LC powders and can also potentially be applied in the preparation of other fluorite type oxides. The spheroidization results showed that the content of solid, defect-free spherical particles was higher in the case of LC powder synthesized by the modified Pechini method. This indicates that the spheroidization ability and efficiency were better for LC powder fabricated by the modified Pechini method than for powder fabricated by solid-state synthesis.

## Figures and Tables

**Table 1:** Crystallite sizes, lattice parameters and chemical composition of the LC powders calcined at 1400 °C.

Sample	Crystallite Size (nm)	Lattice Parameter a (nm)	La (at %)	Ce (at %)	O (at %)
LC-SG	98	0.55729	18.4	18.8	62.8
LC-SS	57	0.55712	18.7	18.9	62.4

SG—modified Pechini sol-gel method, SS—solid-state synthesis.

## Data Availability

Not applicable.
